# Calcific Tendonitis of the Rotator Cuff: An Unusual Case

**DOI:** 10.1155/2012/806769

**Published:** 2012-03-24

**Authors:** Yasuhiro Mitsui, Masafumi Gotoh, Ryo Tanesue, Isao Shirachi, Hideaki Shibata, Kenjiro Nakama, Takahiro Okawa, Fujio Higuchi, Kensei Nagata

**Affiliations:** ^1^Department of Orthopedic Surgery, Kurume University Medical Center, 155 Kokubu-machi, Kurume, Fukuoka 839-0863, Japan; ^2^Department of Orthopedic Surgery, Kurume University, 67 Asahi-machi, Kurume, Fukuoka 830-0011, Japan

## Abstract

Few case reports have described the surgical treatment of calcifying tendonitis of the subscapularis tendon. We present a case of symptomatic diffuse calcifying tendonitis involving the subscapularis and infraspinatus insertions that was difficult to detect arthroscopically. The patient was treated with arthroscopic incision of the tendinous insertions thorough removal of the calcific deposits and subsequent repair using a suture-anchor technique. Two years after the surgical procedure, the patient was completely pain-free and attained full range of motion. Radiographic evaluation performed 2 years after the procedure revealed no calcific deposits. We conclude that the combination of incision of the subscapularis and infraspinatus insertions, complete removal of the calcific deposits, and subsequent suture-anchor repair in an all-arthroscopic manner can lead to an excellent clinical outcome without compromising the functional integrity of the rotator cuff tendons.

## 1. Introduction

Calcific tendonitis of the rotator cuff can be classified into acute, subacute, and chronic types, depending on the time of its onset [1]. Conservative treatment is usually effective for the management of acute and subacute types; however, in patients with the chronic type, the disorder may become resistant to treatment over time. Such patients are treated with arthroscopic surgical removal of the calcifications, which has been reported to be effective [2, 3]. Ark et al. reported good results in 91% of 23 patients with calcific tendonitis of the rotator cuff who underwent arthroscopic surgery [2]. In most cases, calcific tendonitis of the rotator cuff develops in the supraspinatus tendon and rarely affects the subscapularis tendon [4]. We present an unusual case of calcific tendonitis involving the subscapularis and infraspinatus tendons that was treated with an arthroscopic approach.

## 2. Case Report

**Figure 1:**
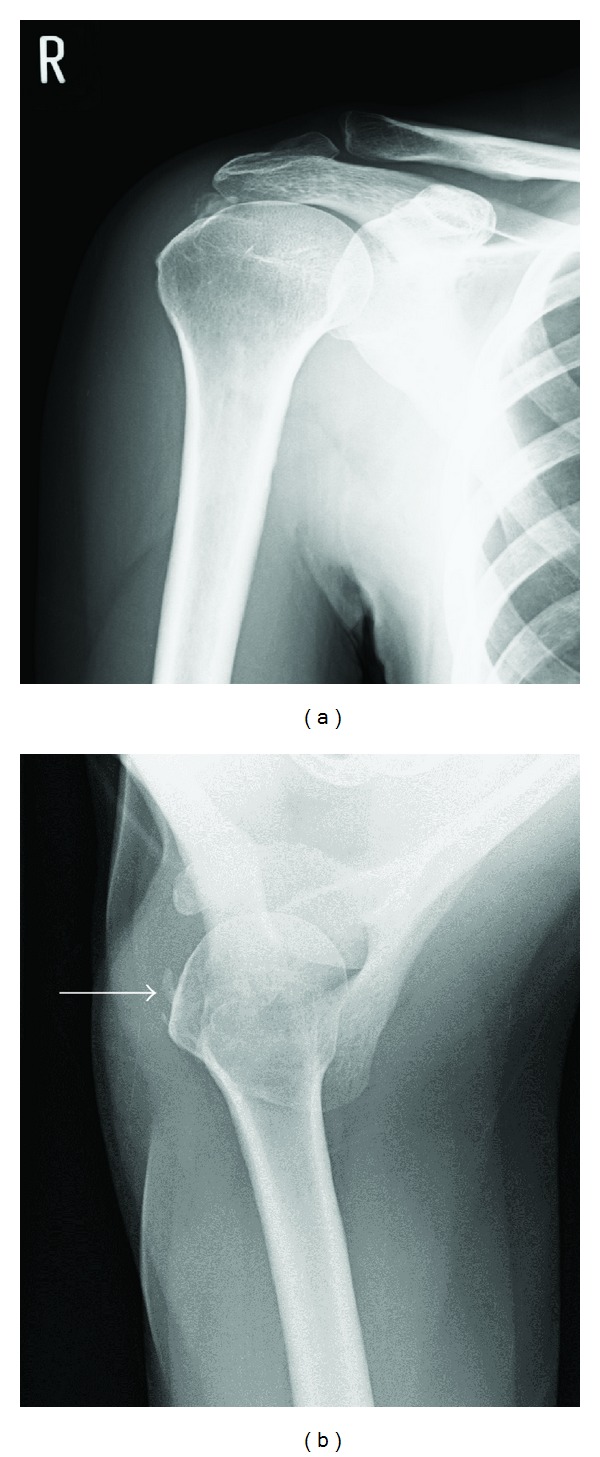
(a) and (b) Preoperative radiographs (anteroposterior view, axillary view) showing calcific deposits in the upper and anterior portion of the humerus. White arrow indicates the presence of calcific deposit at the lesser tuberosity.

**Figure 2:**
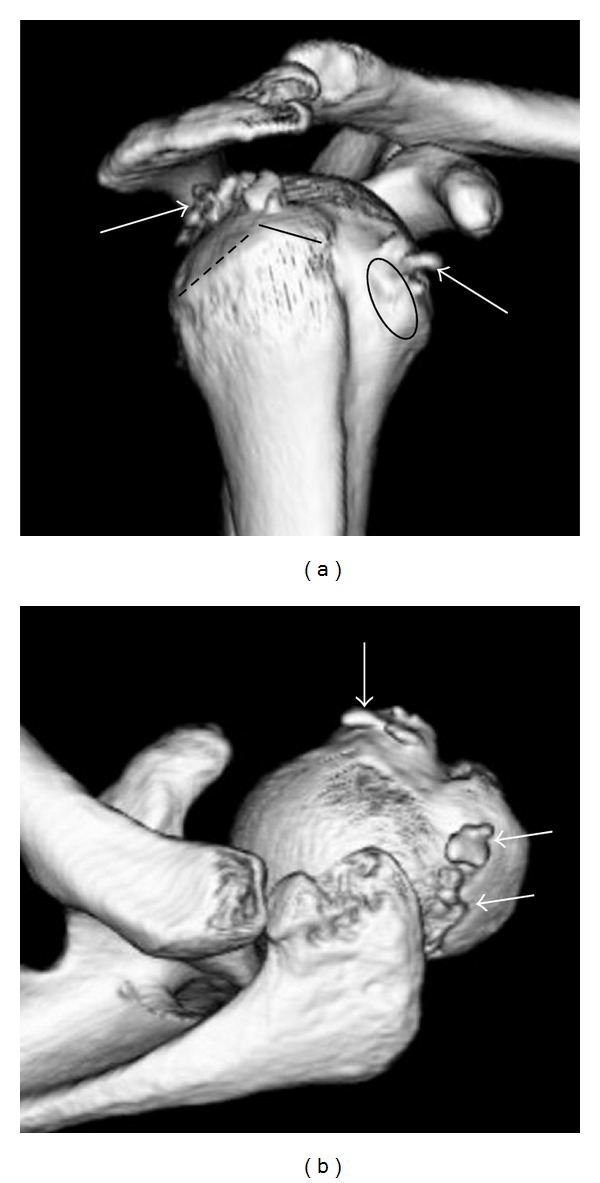
(a) and (b) Three-dimensional computed tomographs showing calcific deposits at the lesser tuberosity and the middle facet of the greater tuberosity. White arrows indicate the presence of calcific deposit. Solid line: superior facet, broken line: middle facet, circle: lesser tuberosity.

A 51-year-old woman who had received conservative treatment (nonsteroidal anti-inflammatory drugs, physical therapy, and subacromial steroid injections) for 2 years for right shoulder pain was referred to our hospital because the shoulder pain was refractory to treatment. At the initial visit, on palpation, tenderness was noted over the lesser and greater tuberosities. Clinical evaluation revealed positive impingement signs [5, 6]. Test of the subscapularis musculotendinous unit, including the belly-press test and the lift-off test, was positive [7, 8]. The patient had nearly full active range -of -motion, but had a painful arc between 60° and 120°. The pain was increased on passive external rotation. Blood tests did not reveal any abnormal findings. Plain radiography showed calcified deposits in the upper and anterior parts of the humerus (Figures 1(a) and 1(b)). Three-dimensional computed tomography (3D-CT) showed calcific deposits at the lesser tuberosity and the middle facet of the greater tuberosity (Figures 2(a) and 2(b)). On the basis of these findings, we made a diagnosis of calcific tendonitis of the subscapularis and infraspinatus tendons and suggested arthroscopic removal of the calcific deposits.

The patient was placed in the beach-chair position under general anesthesia. Standard diagnostic arthroscopy was performed through a posterior portal. Despite careful exploration of the glenohumeral joint and subacromial bursa by using probes, it was difficult to locate the calcific deposits. Therefore, to locate and completely remove the calcific deposits, we decided to detach the subscapularis and infraspinatus tendons from their insertion. The distance between the bicipital groove and the calcifications was initially predicted on the basis of the aforementioned 3D-CT findings. Then, 2 incisions were made at the lesser and greater tuberosities by using the tendon of the long head of the biceps brachii as a landmark. Consequently, complete rotator cuff tears were made at 2 sites. After thorough removal of the calcific deposits, the cuff tear was firmly repaired using the suture-anchor technique.

The patient wore a sling for 6 weeks. Pain-free passive range of motion exercise started immediately from day 1 after surgery. After 6 weeks, active range of motion exercise was permitted, and a muscle-strengthening program was initiated 9 weeks after surgery.

**Figure 3:**
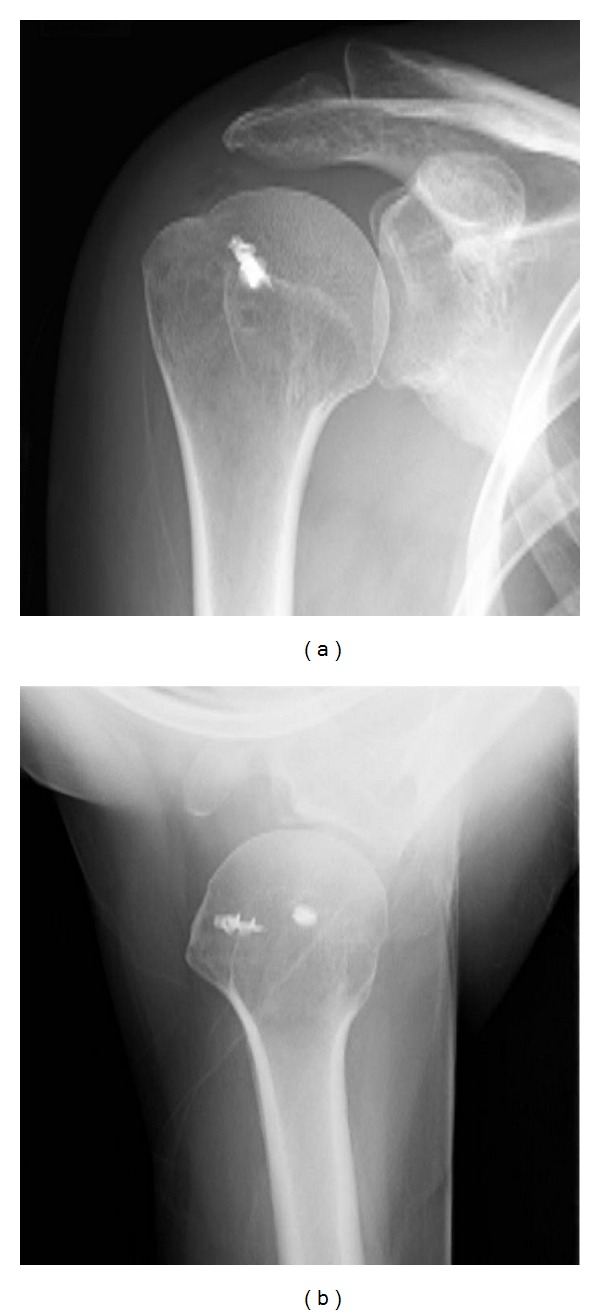
(a) and (b) Postoperative radiographs (anteroposterior view, axillary view) showing the subsidence of the calcific deposits.

Radiography performed 2 years after surgery revealed no calcifications, and the patient showed significant improvement with resolution of pain and improvement in the range of motion (Figures 3(a) and 3(b)).

## 3. Discussion

Although calcific tendonitis most frequently occurs in the supraspinatus tendon, it can also involve more than 1 tendon [9, 10]. However, the frequency of the calcific tendinitis in the subscapularis tendon is low [4, 11, 12]. In the present case, diffuse calcific deposits occurred at the subscapularis and the infraspinatus insertions. To the best of our knowledge, no case of calcifications has been previously reported.

When diffuse calcified deposits are present, as in the present case, determination of the precise locations of the calcifications during arthroscopic surgery may be difficult. Manaka et al. performed 3D-CT before arthroscopic surgery to localize the calcifications; they found it to be very useful for identifying the location of the deposits preoperatively [13]. Since the calcified deposits were diffusely present in an extensive area, preoperative prediction of its location by using 3D-CT was time saving.

Although intraoperative use of fluoroscopy is a valuable method for the localization of calcific deposits, 2 major disadvantages should be considered when using this technique [14]. First, fluoroscopy uses ionizing radiation, and occasionally, patients and staff are exposed to radiation for a long time. Second, difficulties may occur when visualizing the deposit with an intraoperative arthroscope positioned in the subacromial space, particularly when small devices are used.

Recently, Sabeti-Aschraf et al. showed the advantages of intraoperative ultrasound (US) use [14]. They stated that their technique did not increase the risk of joint contamination since the transducer was placed on the skin and did not enter either the subacromial space or the articular space. Although this may be a useful procedure, it remains to be confirmed in the future.

Soft-tissue needle-sparing and intraoperative use of fluoroscopy is a conventional method for removing calcific deposits; however, it would have been difficult in the present case since the calcific deposits were diffusely and extensively present in the tendons. This technique would also have resulted in a large rotator cuff tear and long exposure to ionizing radiation. Manaka et al. reported that after the removal of the calcific deposit, the rotator cuff tear should be sutured when it is greater than 10 mm in size; otherwise, it may further increase in size [13]. Furthermore, Porcellini et al. reported that the number and size of residual calcified deposits were negatively correlated with postoperative results, suggesting a preference for complete removal [15]. In view of these findings, we performed complete removal of the calcifications through a surgical incision at the subscapularis and infraspinatus insertions, ensuring that no residual calcified deposits remained, and subsequently repaired the detached tendons by using the suture-anchor technique.

A cadaveric study confirmed that the upper portion of the subscapularis tendon runs intra-articularly [16]. Ifesanya and Scheibel reported a case of calcific tendonitis in the subscapularis tendon treated through a glenohumeral approach [12]. Although this technique is useful for cases in which the calcifications can be observed intra-articularly, in the present series, we were not able to localize the calcific deposits by glenohumeral inspection. In such cases, the subacromial approach may be employed.

We have presented an unusual case of calcific tendonitis occurring at the subscapularis and the infraspinatus insertions in which surgical detachment and subsequent reattachment were performed using an all-arthroscopic technique. When the calcifications are diffusely scattered over a wide area, our procedure may offer a treatment option for rotator cuff tendonitis.
